# Hypoglycemia post bariatric surgery: drugs with different mechanisms of action to treat a unique disorder

**DOI:** 10.20945/2359-3997000000598

**Published:** 2023-02-07

**Authors:** Giovanna Braganholo Carpentieri, Sandra Elisa Adami Batista Gonçalves, Walid Mohamed Mourad, Lara Guimarães Carelo Pinto, Maria Teresa Zanella

**Affiliations:** 1 Universidade Federal de São Paulo Divisão de Endocrinologia e Metabolismo Departamento de Medicina São Paulo SP Brasil Departamento de Medicina, Divisão de Endocrinologia e Metabolismo, Universidade Federal de São Paulo, São Paulo, SP, Brasil; 2 Universidade Santo Amaro São Paulo SP Brasil Universidade Santo Amaro, São Paulo, SP, Brasil

**Keywords:** Postprandial hypoglycemia, gastric bypass, bariatric surgery

## Abstract

Postprandial hypoglycemia (PPH) is a complex and multifactorial complication of bariatric surgery (BS). PPH may cause severe symptoms or be asymptomatic. The treatment of this condition requires dietary changes, but severe cases require drug therapy. The number of therapeutic options is limited and are often associated with adverse side effects. Different classes of drugs have been used and tested, but the resolution of PPH remains a challenge for physicians and patients. In this review, we gathered articles on PPH after BS from PubMed searches (2001 to 2022) and focused on the main drugs tested for the treatment of this condition, such as acarbose, somatostatin analogues, type 2 sodium-glucose cotransporter inhibitors, calcium channel blockers, and liraglutide. Avexitide and glucagon pump are two new therapeutic options that have been recently tested. For the search, the terms “postbariatric hypoglycemia,” “bariatric surgery,” and “late dumping syndrome” were used. PPH after BS is a frequent condition that should always be evaluated after BS. Treatment should be individualized and the available therapeutic options may be useful based on the condition's pathophysiology.

## INTRODUCTION

Bariatric surgery (BS) is currently the most effective treatment for severe obesity in terms of weight loss and improvement of obesity-related comorbidities (1,2). Although safe, BS has complications that have increased with the marked rise in the number of BSs performed worldwide (2,3). Postprandial hypoglycemia (PPH) is a complication that usually manifests within 1 to 3 hours after a high-carbohydrate meal and presents with improvement of neuroglycopenic and adrenergic symptoms after glucose ingestion (4,5). The risk factors for this condition are lower age, female sex, greater loss of excess body weight after surgery, and higher beta-cell function (6,7).

Hypoglycemia is a consequence of anatomical and physiological changes in the gastrointestinal tract following food consumption and the metabolic repercussions the changes cause, but the pathophysiology is still not fully understood. After a meal, ingested nutrients pass quickly into the intestine, leading to earlier spikes in blood glucose, when compared to nonsurgical patients. It has been proposed that this would result in inappropriately elevated levels of type 1 glucagon-like peptide (GLP-1) and insulin, which, in association with possible reductions in the release of counter-regulatory hormones, could cause episodes of PPH (8-10).

Symptoms diverge among patients and range from asymptomatic hypoglycemia to debilitating symptoms that worsen quality of life and increase the risk of dementia and death from all causes (11). Some patients may have multiple episodes of hypoglycemia per day, in a cycle in which symptoms are manifested after a high-carbohydrate meal, followed by glucose ingestion with improvement in symptoms and a new glycemic peak with subsequent hypoglycemia, characterizing the recurrent pattern as a “rollercoaster” (12).

A recent study using continuous glucose monitoring (CGM) found that the prevalence of PPH after BS is higher than described in previous studies and can occur in up to 50% of patients undergoing Roux-en-Y gastric bypass (RYGB) and vertical sleeve gastrectomy (13).

No consensus has been established for the diagnostic criteria for PPH after BS, so patients with symptoms such as Whipple's triad (low glucose values associated with symptoms or signs and relief of symptoms when glucose levels are raised) should be investigated (14,15). Tools that can be used include questionnaires (7), mixed or liquid meal tolerance test (MTT) (16), and CGM (17). Although CGM has low accuracy in detecting low glucose levels, it can be useful to identify patterns of glycemic excursions, but not for diagnosis (14,15). Recently, the detection with CGM of nocturnal episodes of hypoglycemia in these patients has been observed (13,17). The oral glucose tolerance test (OGTT) has been used in RYGB patients since the 1980s (10), but this method is poorly tolerated in patients undergoing BS because hyperosmolar fluid overload can cause severe dumping symptoms, therefore it should be avoided (14,15). The MTT is the preferred provocative test for the diagnosis of PPH after BS (10).

The treatment of this condition is challenging and involves dietary changes (18) and medications in refractory cases, but a few therapeutic options are effective (19). The drugs studied for the treatment of PPH after BS differ in the mechanism of action by which they improve hypoglycemia. Acarbose (20,21) and canagliflozin (22) would act by slowing the absorption of carbohydrates. Calcium channel blockers (21,23), diazoxide (24,25), somatostatin analogues (26,27), and GLP-1 agonists (28,29) and antagonists (8,30) would act by reducing the postprandial insulin peak.

In cases of severe PPH that are refractory to medication, surgical techniques have been used, such as gastrostomy in the excluded stomach (31), placement of a silicone ring in the gastric pouch, bypass reversal with and without concomitant sleeve resection, and distal pancreatectomy (32).

Each of these interventions is a possible therapeutic option for the treatment of PPH after BS and they must be evaluated individually. Therefore, this review on clinical treatment aims to assess recommended dietary recommendations and to describe the main mechanisms of action, as well as adverse events, of the drugs already tested in the treatment of these patients (10,14).

### Dietary recommendations

Once the diagnosis has been made, the first step in the management of PPH is dietary counseling, which is the mainstay of treatment for all patients, primarily aimed at reducing the frequency of hypoglycemic events (12).

Controlling the carbohydrate intake of each meal by restricting the maximum consumption to 30 g of carbohydrates per meal and 15 g per snack is recommended. Due to differences in glucose absorption at the intestinal level of each patient, some do not tolerate this amount, and therefore individualization of guidance is necessary as well. Patients can use apps or manuals for carbohydrate counting (33,34).

The type of carbohydrate chosen is another important factor in choosing the foods to consume. A low glycemic index (GI) diet is desired to reduce the postprandial blood glucose peak and thus avoid causing an exaggerated stimulus for insulin secretion. The GI classifies food according to the postprandial glycemic response produced by a carbohydrate, which can be classified as low GI (≤55%), medium GI (56%-69%), or high GI (≥70%) (35). Low GI carbohydrates are digested more slowly and they reduce the peak blood glucose after meals and are therefore more suitable. Foods with fiber and fat tend to have a lower GI and they are more recommended (18).

The inclusion of fats immediately before meals and snacks, which normally delays gastric emptying, does not significantly affect postprandial hyperglycemia because gastric emptying in these patients occurs rapidly, but may help as a source of calories to replace carbohydrates. Protein ingestion before meals can be useful because it acts by reducing intestinal glucose absorption, increasing early satiety, and potentiating the incretin response (36). Eating 60 to 120 g of protein a day or 0.9 g of protein per kilogram of current weight is recommended (37).

Physical activity can exacerbate hypoglycemia and in patients who present symptoms during exercise. Measuring capillary blood glucose before exercise and in case of blood glucose below 80 mg/dL is suggested. A 15-g portion of low GI carbohydrate associated with 5 to 8 g of fat must be ingested before beginning the activity (18).

### Acute treatment of hypoglycemia

In hypoglycemia, treatment requires rapid ingestion of 15 g of carbohydrates (*e.g.*, four glucose tablets or one tube of glucose gel) (18). Because simple carbohydrates can have a rebound effect with new hypoglycemia, a fat or protein can be associated with the correction of mild episodes of hypoglycemia (14). In severely hypoglycemic patients with neuroglycopenic symptoms, subcutaneous glucagon may be applied.

### Preventive drug treatment

**Figure 1:**
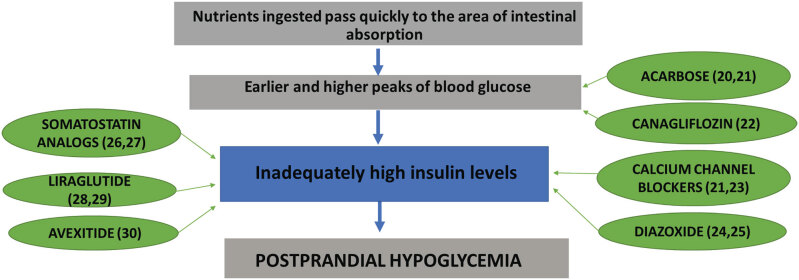
Pathophysiological mechanisms of PPH after BS and mechanisms of action of different therapeutic agents

If nutritional measures are not sufficient to improve symptoms in patients with PPH, then drug treatment should be initiated. Some of the most used medications for treating PPH were compared in a recent crossover study randomized with 11 patients who had hypoglycemia (38). The drugs tested were acarbose, sitagliptin, verapamil, liraglutide, and pasireotide. It was concluded that only acarbose, by decreasing the postprandial peak of insulin and pasireotide and reducing the postprandial peaks of insulin and GLP-1, was effective in improving the glucose nadir and preventing PPH in patients (38). Medications act on different mechanisms involved in the pathophysiology of PPH. Figure 1 correlates the action of each medication to the pathophysiology of PPH after BS.

The main mechanisms involved in the treatment of PPH will be described for each medication already used, according to the type of study in which the medications were tested and their clinical evidence. Table 1 summarizes the main medications used to treat PPH.

**Table 1:** Summary of the main medications already used in the treatment of PPH after BS

MEDICATIONS USED FOR THE TREATMENT OF PPH EFFECTIVELY IN CLINICAL TRIALS
ACARBOSE (20,21)
• Mechanism of action: delays the absorption of carbohydrates, reducing postprandial glycemic and insulinemic peaks.
• Dose: 100 to 300 mg/day before main meals. Start with 25 mg/day and increase gradually
• Side effects: flatulence, diarrhea, abdominal pain.
SOMATOSTATIN ANALOGS (OCTREOTIDE AND PASIREOTIDE) (26,27)
• Mechanism of action: reduces GLP-1 levels and inhibits insulin secretion
• Dose: octreotide 25 to 50 ug SC before meals; pasireotide SC 75 to 300 mcg/day
• Side effects: diarrhea, steatorrhea, abdominal pain, gallstones, QT interval prolongation, persistent hyperglycemia
CANAGLIFLOZIN (22)
• Mechanism of action: Canagliflozin: reduces carbohydrate absorption by inhibiting intestinal SGLT-1 and thus decreases postprandial insulin spike
• Dose: 300 mg per day
• Side effects: genital infections, volume depletion, increased urinary frequency
AVEXITIDE (currently in phase II of testing) (30)
• Mechanism of action: GLP-1 receptor antagonists, reduce postprandial insulin and GLP-1 secretion and increase glucagon
• Side effects: Headache, nausea, and subcutaneous injection site reaction.
**MEDICATIONS ALREADY USED, HOWEVER, ONLY CASE REPORTS SHOW EFFECTIVENESS**
CALCIUM CHANNEL BLOCKERS (VERAPAMIL (21) AND NIFEDIPINE (23))
• Mechanism of action: reduce postprandial insulin secretion
• Dose: verapamil: 80 mg twice a day; nifedipine: 30 mg/day
• Side effects: water retention, hypotension, edema, nausea, headache
DIAZOXIDE (24,25)
• Mechanism of action: activates ATP-dependent potassium channels and decreases the opening of calcium channels, reducing insulin release
• Dose: 50 mg, 2x/day
• Side effects: fluid retention, hypertrichosis, gastrointestinal disorders, edema and neutropenia
LIRAGLUTIDE (28,29)
• Mechanism of action: persistent activation at the GLP-1 receptor leading to inhibition of insulin release and increased glucagon release in hypoglycemic conditions
• Dose: 0.6 mg SC/day initially, progress to 1.2 mg SC
• Side effects: nausea, headache, stomach pains, constipation, diarrhea

### Medications with proven efficacy in clinical trials

#### Acarbose

Acarbose is an on-label antidiabetic drug that acts as a competitive inhibitor of intestinal alpha-glucosidase by blocking the hydrolysis of polysaccharides, oligosaccharides, and disaccharides into monosaccharides, which results in a reduction in the rate of absorption of dietary carbohydrates. With this action, it is possible to delay and attenuate the elevation of plasma glucose, which consequently can reduce the peak serum insulin (20).

Valderas and cols. (20) found that in eight patients with symptomatic hypoglycemia after RYGB, the administration of 100 mg of acarbose 15 minutes before meals was sufficient to prevent symptomatic hypoglycemia (46.4 ± 4.8 *vs.* 59.0 ± 2.6 mg/dL, *p* < 0.01). In addition, the treatment promoted a reduction between the peak and the nadir of postprandial glycemia and reduced the areas under the curve of glycemia and insulin (20). Similarly, Ritz and cols. treated eight patients with PPH after RYGB with acarbose 50 to 100 mg three times a day, before the main meals, in association with dietary modifications and they observed elimination of symptoms and improvement in the glycemic profile during CGM after meals (39).

The action of acarbose ends up increasing the load of unhydrolyzed carbohydrates in the intestinal lumen, which can trigger symptoms of intolerance such as flatulence and abdominal distension. Therefore, it is suggested that the dose of acarbose be gradually increased to the maximum tolerated dose (*e.g.*, begin with 25 mg and increase the dose gradually, up to 100 mg three times a day). Administering the dose before the main meals is also suggested to act more specifically on the absorption of carbohydrates (20). If hypoglycemia does occur while taking acarbose, it should be corrected with glucose, dextrose, honey, or milk because the options typically used to treat hypoglycemia (*e.g.*, table sugar, juice, regular soft drink, or sugar candy) will not be effective considering the type of the action of the drug.

#### Somatostatin analogues

Somatostatin analogues are on-label drugs used for the treatment of gastroenteropancreatic neuroendocrine tumors and exert several effects that may influence intestinal motility and alter the release of several gut hormones that are relevant in the pathophysiology of hypoglycemia (40). First, in normal individuals, octreotide delays antral contractility and therefore delays gastric emptying. It also inhibits postprandial secretion of several vasoactive and metabolic hormones, mainly gastrin, insulin, and cholecystokinin. However, somatostatin analogues exert their therapeutic effect by inhibiting insulin and GLP-1 secretion to prevent postprandial hypoglycemia (26).

It was shown that the subcutaneous administration of octreotide 50 to 100 mcg before meals was able to improve symptoms related to early and late dumping syndrome after gastric or esophageal surgery, in addition to preventing late hypoglycemia. This was due to its action of reducing the postprandial insulin spike and prolonging the rise in plasma glucose (40). Both subcutaneous daily octreotide and long-acting formulations of somatostatin analogues (Sandostatin LAR 20 mg intramuscular monthly) significantly reduced total dumping severity scores in patients with early and late dumping syndrome after gastrointestinal surgeries, as shown by Arts and cols., although they found a significant improvement in quality of life in the monthly administration (26). Pasireotide, a somatostatin analogue with strong inhibition of insulin secretion, was tested in five women with PPH after BS at doses of 75, 150, and 300 mcg, with the pasireotide injections being given subcutaneously 30 minutes prior to meal intake. It was observed that all doses prevented postprandial hypoglycemia, but also led to important increases in hyperglycemia, probably caused by the strong reduction in the postprandial insulin and GLP-1 response. It was concluded that the best therapeutic option would be the dose of 75 mcg, but pasireotide should be used with caution in these patients due to the possible risk of persistent hyperglycemia. Another problem with this class of medications is their potential to cause gallstones, abdominal pain, steatorrhea, and QT prolongation. It is estimated that about half of patients may have their treatment interrupted due to adverse events, mainly digestive symptoms such as abdominal pain and flatulence (27).

#### Canagliflozin

Canagliflozin, an on-label antidiabetic drug, has recently been tested for the treatment of PPH after RYGB in a pilot study. Twenty-one patients with PPH were evaluated and the response to the OGTT was compared before and after the use of canagliflozin 300 mg for 2 weeks. The study showed patients taking the medication had a significant reduction in glucose and insulin peak after glucose overload, and a reduction of 85.7% in hypoglycemic episodes was observed after 180 minutes. The authors suggested that canagliflozin would reduce the intestinal glucose absorption through inhibition of intestinal type 1 sodium-glucose cotransporter (SGLT1). In this study, short-term canagliflozin therapy was not associated with adverse events (22).

### Medications already used, but only case reports show efficacy

#### Diazoxide

Diazoxide is an antihypertensive medication, but it can also be used to treat hypoglycemia caused by insulinoma. As an activator of potassium channels that hyperpolarize cells (especially pancreatic beta cells) and therefore inhibit calcium channels, diazoxide reduces insulin secretion. Studies using diazoxide for the treatment of PPH after BS are rare, but a recent publication has reported its use to treat a patient who had hypoglycemia after RYGB refractory to treatment with acarbose and was not tolerant to the gastrointestinal effects caused by octreotide, which was discontinued due to diarrhea. After the use of diazoxide 100 mg twice a day, the patient had an improvement in neuroglycopenic symptoms and hypoglycemia, without any side effects (25). The main side effects described using diazoxide over time are hypertrichosis, water retention, gastrointestinal symptoms, edema, and neutropenia. In principle, its use for the treatment of PPH after BS is suggested for cases whose treatment was refractory to dietary interventions and conventional drugs (24).

#### Calcium channel blockers

Calcium channel blockers (CCBs) can block insulin secretion by inhibiting calcium channels in pancreatic beta cells. Therefore, they can potentially reduce the hypoglycemia that occurs after an insulin spike. Moreira and cols. first reported the case of a successfully treated patient who had improvement of clinical symptoms with the combination of verapamil and acarbose (21). Years later, Ames and cols. reported two cases in which they obtained good responses with the use of nifedipine or verapamil in PPH after BS, after failure of conventional treatment with acarbose, diazoxide, and octreotide (23). These reports demonstrate that this class of drugs may represent an alternative for symptomatic PPH that is refractory to dietary interventions and other drugs, although its efficacy has not been demonstrated in a clinical trial (38).

#### Liraglutide

Liraglutide is an on-label GLP-1 analogue used for treatment of diabetes and currently for obesity. Abrahamsson and cols. treated five consecutive patients submitted to RYGB who presented PPH symptoms with subcutaneous liraglutide dosage from 1.2 mg to 1.8 mg daily. All patients reported disappearance of symptoms with the use of liraglutide, but symptoms relapsed for four of the five patients after the discontinuation of the treatment (28). Chiappetta and cols. reported the use of liraglutide 1.2 mg in the treatment of PPH refractory to dietary and drug interventions with acarbose and somatostatin analogues; in this case the patient had undergone Toupet fundoplication for the treatment of gastroesophageal reflux. Synchronization between insulin and glucose secretion occurred with the use of liraglutide, with the glucose peak preceding the insulin peak, in such a way that the abrupt drop in blood glucose was delayed. The authors suggested that the use of exogenous GLP-1 would keep the receptors activated, which could explain the lower levels of total insulin and resolution of symptoms (29). The side effects reported were nausea, headache, stomach pains, heart flutters, and diarrhea (28).

In the randomized crossover study performed by Øhrstrøm and cols., in which liraglutide was compared to other commonly used medications such as acarbose and pasireotide, no reduction in hypoglycemia was seen after the MTT, but the findings suggest an effect as a blood glucose stabilizer (38).

### Medications in clinical investigation

#### Avexitide

GLP-1 receptor antagonist drugs are strong candidates for the drug treatment of PPH because the inadequate secretion of GLP-1 in response to the rapid passage of food to the intestine plays an important role in its pathophysiology (8). In the first study with continuous infusion of GLP-1 receptor (GLP1R) antagonist, exendin 9-39, nine patients with PPH were submitted to mixed-meal tolerance test and all had their hypoglycemia corrected by administration of the GLP1R antagonist. During the study, patients with PPH had a longer period until they reached the blood glucose nadir, not reaching levels below 50 mg/dL (8).

Years later, a phase II trial revealed efficacy of the use of twice-daily lyophilized avexitide (exendin 9-39) by subcutaneous injection for 3 days in 19 patients with PPH after BS. This medication was efficient in reducing postprandial hyperinsulinemia and it raised the glucose nadir, preventing severe hypoglycemia after the OGTT (30). No significant side effects were reported during the use of the medication in the studies, indicating that the drug appears to be an effective, well-tolerated, and safe option due to its action on one of the main mechanisms of the pathophysiology of PPH (8,30).

#### Glucagon pump

Still under clinical investigation, the use of glucagon in an automated closed-circuit pump has shown satisfactory results. The system has shown to be very promising for trying to bring improvements in the quality of life and safety of patients with PPH (41). Lobato and cols. suggested that glucagon secretion preceding the insulin peak is important to prevent hypoglycemia after a mixed meal test. This reinforces the idea that glucagon prepares the liver to maintain sustained glucose production when its peak occurs within 15 minutes of a meal (42).

A double-blind, placebo-controlled study analyzed 12 patients with PPH and tested glucagon versus placebo using an automated closed-loop pump. The glycemic nadir was higher in patients who received glucagon, and no episodes of hypoglycemia were reported. The 12 participants reported mild discomfort at the site of application of both glucagon and placebo and no other adverse effects were observed (41).

The purpose of this new technology is based on the system's ability to detect hypoglycemic episodes very quickly. Glucagon is released according to a hypoglycemia algorithm that has been incorporated into an artificial pancreas system guided by continuous monitoring of serum glucose levels. By releasing glucagon into the bloodstream, the system would enable corrections of hypoglycemia by mobilizing hepatic glycogen, which is released into the bloodstream in the form of glucose, promoting reversal of the hypoglycemic condition (43).

In conclusion, PPH is a frequent complication after BS and its treatment should always begin with nutritional measures that improve symptoms in most patients. For patients who are refractory to dietary changes, some medications have been shown to be effective in randomized trials, such as acarbose, somatostatin analogues, and canagliflozin. Other medications such as CCBs, diazoxide, and liraglutide have been shown to be effective only in case reports, but they should not be excluded from the therapeutic arsenal because some patients could experience clinical improvement. GLP-1 antagonists are being studied, and research to date points to the possibility that they may become another therapeutic option for the challenging treatment of PPH after BS.
